# Loss of Smell and Taste: Potential of Using Them as Markers for Early Detection of Covid-19

**Published:** 2020-04-26

**Authors:** Pinky Kain

**Affiliations:** Laboratory of Neuroenetics, Regional Centre for Biotechnology, NCR Biotech Science Cluster, Faridabad, Haryana, India

The world is currently witnessing a severe health crisis of its time. Everyone is juggling and struggling to fight a viral disease named Covid-19 (Corona virus disease 2019) caused by SARS-CoV-2 (Severe Acute Respiratory Syndrome Coronavirus 2). The genome of newly identified virus is less than 30,000 letters long where pathogenesis involve from being asymptomatic to deadly in all age groups. Almost everybody is at a grave risk in such an unpredictable scenario where range of symptoms are so variable from people to people making this pandemic a threat to human race.

Despite having respiratory innate immunity and other protective barriers including secretion of mucus in the upper respiratory tract, the incidences of infection in this part of the body is a huge economic burden on a society and are the key factors for developing antibiotic resistance. Infections in the upper respiratory tract by viruses are not new and have been well documented for ages. Many viruses can lead to such infections including the common cold virus. Among many symptoms that a patient suffering from viral infection experience, the partial or complete loss of smell and taste are lesser known symptoms of many viral diseases including Covid-19. Such symptoms have been raised and reported recently by British Association of otorhinolaryngology (ENT UK). These symptoms have been found in many patients in the absence of other obvious symptoms which includes continuous cough with high temperature, fatigue and respiratory issues. There is a rise in number of isolated anosmia and hyposmia which could be complete or partial loss of smell in asymptomatic patients. Large number of cases have been reported in China, South Korea, UK, USA, northern Italy and France. Until now such patients are potential silent and hidden carriers which can easily escape testing or isolation criteria. Alterations in olfactory behavior also affects the taste behavior, often both are associated and it is difficult to separate the two in the absence of one. Nonetheless, absence of taste (parosmias and/ or dysgeusias) or smell or both could cause serious health problems for such carriers if not the virus per se. Unable to sense the right temperature, toxins and harmful chemicals in the surrounding may cost their lives. It won’t be surprising if the symptomatic patient experience the same more strongly during the course of the infection and face difficulty in appreciating the taste and smell of food or medication they are on.

Post viral loss of smell and taste could be a result of blockage of receptor sites, loss of receptor function, inflammation of olfactory nerves, injury of the specialized nerve tissue, falling of dendrites receiving the signal, alterations in the higher smell pathways in the brain or change in the sensitivity of the receptor neurons in the nasal tissue due to congestion that causes symptoms like cough and running nose.

Depending on the route of entry, the viral load could be high in the pharynx, nose and throat or each of these organs. Virus can attach to different cells of these organs possibly and cause transient loss of smell in patients that last anywhere between 7 to 14 days. Data suggesting the exact duration of getting the chemosensory powers back between symptomatic and asymptomatic patients is still not sufficient. Slow improvement rates could be discouraging, but with the resumption of nasal breathing, chemosensory function should reappear too with time.

Temporary loss of smell and taste due to congestion in case of Covid-19 is like any other virus like symptoms. It is only recently these symptoms have been considered important and useful as potential markers to find the asymptomatic patients. Due to the duration of treatment and severity of the disease, a small percentage of patients may suffer from persistent chemosensory loss too. In people suffering from cold or sinusitis due to seasonal changes would be difficult to discern between Covid-19 viral damage and an ongoing process in the nose as the cause of the loss. Such patients should opt for other methods of testing and make sure they are not suffering from covid-19.

Although taste receptors were initially found in the oral epithelium, new data suggest they are distributed more widely in the body including gastrointestinal epithelium, brain, thyroid and upper and lower respiratory tracts. The presence of extra-oral taste receptors in nasal epithelium suggest their role as respiratory innate defenses and may play a role in the detection of invading respiratory pathogens [1]. Future studies should focus on looking at their potential therapeutic use and help clinician to decrease the economic burden of upper respiratory tract diseases. Modulation of taste receptors present in the nasal epithelium could be used for the targeted therapies in the treatment of infections of upper respiratory tract specially at the time of pandemic.

Can loss of smell or taste be considered defining features of the disease are still under debate? Since olfactory and taste disorders are common with advanced age, there is a need to separate the data based on the age and get a clear picture about who actually is suffering from it in case of covid-19. No data clearly suggest whether it is the whole covid-19 infected population or only certain cases are showing loss of smell and taste symptoms including the asymptomatic carriers. Setting up of studies on people tested for the virus could give a clear picture.

Many parameters can explain and significantly affect different outcomes in patients suffering from Covid-19 including genetics, immunological history and current immune system state, viral load causing the infection, the path of entry into the body (eyes, nose or mouth), virus virulence and frequency of change in its own structure. Although there is currently no available therapy to be effective, and many vaccines are under trial to treat the viral infection, one should consider these symptoms as an emergency and consult a doctor and get tested for the virus as soon as possible.

Use of artificial intelligence (AI) as a method of mass screening of people by accessing their breath and determining what volatiles are inhaled in and exhaled out could help in evaluating illness. Compounds inhaled by healthy person versus infected people could create distinctive fingerprints. Such techniques having potential in wide application in research and medical sciences would be very useful in such a pandemic. Presence of viral load in the nose and its effects on the olfactory neurons may quite change the air that one exhales out. Diagnosing people with AI not only will help in easily separate population with loss of smell but detection of illness would be simple and possible at early stages and can help in taking appropriate measures early enough to cure. Once diagnosed properly, patients can be treated with the best possible management. Such trials using AI would generate data with numbers to inform if loss of smell and taste could be considered as early symptoms in such a pandemic and would help even for the future studies.

With the limitations of testing, it is challenging to get the real numbers suffering from loss of smell and taste due to covid-19. New interventions and vaccines are the need of the hour with highest precautions. One should not use any inhaler or any other drug for local modulation without consulting the doctors to revive their powers of taste and smell. The best suggestion for such patients is to isolate themselves to reduce the spread to infection to others.
